# Academic Resilience in Mathematics, Mathematics Identity, School Engagement and Academic Procrastination in Early Adolescence: A Latent Profile Analysis

**DOI:** 10.3390/bs16101912

**Published:** 2026-10-15

**Authors:** Muhammed Celal Uras

**Affiliations:** Department of Mathematics Education, Faculty of Education, Ağrı İbrahim Çeçen University, Ağrı 04100, Türkiye; mcuras@agri.edu.tr

**Keywords:** academic resilience in mathematics, mathematics identity, school engagement, academic procrastination, early adolescence, latent profile analysis

## Abstract

This study examined how academic resilience in mathematics combines with mathematics identity, school engagement, and academic procrastination, and explored the associations between these configurations and mathematics achievement. Participants were 897 students aged 10–14 years from three public middle schools in eastern Türkiye. Latent profile analysis of standardized scale totals retained five profiles: (1) higher resources, lower procrastination; (2) school engagement, lower mathematics resources; (3) mathematics resources, lower school engagement; (4) mixed pattern, high variability; and (5) lower resources, higher procrastination. Profile sizes ranged from 10.0% to 28.3%. Profile 4 was a heterogeneous, dispersion-driven component, and the profile structure depended on variance–covariance assumptions. The BCH procedure indicated overall differences in student-reported previous-semester mathematics grades, χ^2^(4) = 24.25, *p* < 0.001, with a model-based eta-squared analogue of 0.038. After Holm adjustment, Profile 1 had higher grades than Profiles 4 and 5. Grade-level-adjusted comparisons yielded the same pattern. The findings describe differing combinations of perceived mathematics resources and academic adjustment that may inform investigations of support needs. The cross-sectional design precludes causal conclusions, and the profiles require independent replication.

## 1. Introduction

Early adolescence is an important period for understanding how students respond to mathematics difficulties and sustain their participation in school. Changes in adolescents’ needs for competence, autonomy, and belonging make the fit between students and their learning environments especially consequential (Eccles et al., 1993). In a longitudinal study spanning grades 5–9, Frenzel et al. (2010) documented declining mathematics interest, with substantial differences in students’ levels of interest. Therefore, difficult tasks, disappointing results, and competing school demands warrant attention alongside achievement itself. Research on academic buoyancy highlights the educational relevance of handling such everyday setbacks (Martin & Marsh, 2008). The present study approaches these experiences through the joint patterns of academic resilience in mathematics, mathematics identity, school engagement, and academic procrastination.

### 1.1. Theoretical Framework

A mathematical resilience perspective provides the broader framework. It directs attention to students’ capacity to continue learning when mathematics becomes difficult. Xenofontos and Mouroutsou’s (2023) review of 78 empirical studies illustrates the range of definitions and approaches used in this field. Therefore, conceptual precision is important. Martin and Marsh (2008) distinguish responses to ordinary academic setbacks from resilience in circumstances of substantial adversity. In this study, academic resilience in mathematics is operationalized as students’ perceived coping capacity when facing mathematics difficulties. The measure does not establish resilience in a clinical sense or successful adaptation following independently documented adversity. This interpretation connects the study to coping and academic adjustment while respecting the scope of the measures. Situated expectancy-value theory helps explain why this coping capacity should be considered alongside mathematics identity. The theory relates achievement-related choices and persistence to expectations of success and the value attached to an activity, within students’ developmental and sociocultural contexts (Eccles & Wigfield, 2020). Mathematics identity concerns how students understand themselves in relation to mathematics, including their interest, perceived competence, and recognition by others (Cribbs & Utley, 2024). Thus, it provides a complementary account of the personal meaning of mathematical participation. Its conceptual links with expectancies and values help explain why feeling able to manage difficulty and seeing mathematics as part of oneself may be associated. Nevertheless, identity includes social recognition and should not be reduced to a measure of self-efficacy. Two further perspectives connect these mathematics-specific resources to broader academic behavior. Finn’s (1989) participation–identification model links participation in school with identification with schooling, including belonging and valuing school. This school-level identification is conceptually distinct from identification with mathematics as a subject. Temporal motivation theory addresses the enactment of intentions by integrating expectancy and value with sensitivity to delay and the timing of rewards (Steel & König, 2006). It helps explain why valuing academic success may coexist with postponing the work required to achieve it. These perspectives justify examining perceived coping capacity, mathematics-related self-understanding, school participation, and task delay as complementary features of academic adjustment. They guide the selection of indicators without imposing a causal sequence among them.

### 1.2. Mathematics Resources and Academic Adjustment

#### 1.2.1. Academic Resilience in Mathematics

Academic resilience in mathematics concerns students’ perceived ability to persist and respond constructively when they encounter difficulty in mathematics. The domain-specific assessment developed by Ricketts et al. (2017) makes this capacity accessible to empirical study. Resilience research also emphasizes that its meaning depends on the challenges and educational settings under consideration (Xenofontos & Mouroutsou, 2023). Conceptually, the capacity to respond to a mathematics setback need not coincide with the importance of mathematics in a student’s sense of self. Thus, resilience contributes information about responding to challenge alongside the meaning of participation.

#### 1.2.2. Mathematics Identity

Mathematics identity reflects students’ sense of themselves as mathematics learners. Research with students in grades 5–12 supports an assessment approach encompassing interest, recognition, and competence/performance beliefs (Cribbs & Utley, 2024). These components connect personal evaluations of mathematics with how students believe others recognize their mathematical capabilities. Identity is relevant to the present framework because the meaning students attach to being a mathematics learner may accompany their willingness to remain involved when work becomes demanding. The present study uses a broad identity score to represent this orientation.

#### 1.2.3. School Engagement

School engagement encompasses behavioral participation, emotional connection, and cognitive investment in schooling (Fredricks et al., 2004). A recent meta-analysis of 137 studies found positive associations between engagement and achievement, with differences across engagement dimensions and measurement approaches (Wong et al., 2024). Therefore, engagement contributes information about students’ participation in their wider educational setting. Its inclusion alongside mathematics-specific indicators permits examination of whether resources within mathematics correspond with involvement in school more generally. A broad engagement score summarizes these related dimensions while retaining their multidimensional conceptual basis.

#### 1.2.4. Academic Procrastination

Academic procrastination involves postponing intended academic work despite anticipating disadvantages from the delay. Steel’s (2007) meta-analytic and theoretical review situates procrastination within difficulties in self-regulation. A scoping review focused on children and adolescents further establishes its relevance before university and identifies a need to extend knowledge at earlier educational stages (González-Brignardello et al., 2023). Including procrastination captures difficulties in initiating or completing schoolwork that may remain hidden when a study considers only positive academic resources. It is related to engagement but provides a more specific indication of habitual task delay.

### 1.3. Relationships Among the Study Variables

Empirical findings support several connections within this framework. Among Australian students in years 8 and 10, mathematics-related academic buoyancy was associated with engagement and self-efficacy across two measurement occasions (Martin & Marsh, 2008). Buoyancy is a related construct rather than the same measure of academic resilience in mathematics used here. In a study of 525 sixth-grade students, Miller and Wang (2019) reported associations linking classroom practices, competence beliefs, task values, and mathematics identity. This finding supports considering the motivational and contextual meaning of identity in early adolescence. In a more recent study of 450 Peruvian secondary students, academic engagement and self-efficacy were each negatively associated with procrastination (Cutipa-Flores et al., 2025). These findings provide a basis for studying the four constructs jointly, but they do not establish every pairwise relationship in the present framework. In particular, competence beliefs offer relevant evidence without serving as substitutes for either mathematics identity or academic resilience in mathematics. The proposed integration concerns the possibility that students’ perceived capacity to handle mathematics difficulties and their identification with mathematics may align unevenly with general school engagement and task management. Therefore, examining both resources and procrastination can distinguish broadly favorable adjustment from combinations in which positive self-perceptions coexist with difficulties in academic action. This possibility is a theoretical rationale to investigate, not a predetermined description of the profiles.

### 1.4. A Person-Centered Approach and the Research Gap

Variable-centered analyses clarify the average associations among constructs. A person-centered approach adds a complementary question about how these constructs combine within students. Latent profile analysis (LPA) estimates probabilistic subgroups from continuous indicators and permits differences in the levels and configurations of those indicators (Spurk et al., 2020). LPA has also been used to examine mathematics-related competency profiles in preservice teachers (Uras et al., 2025). It can assess whether mathematics resources, school engagement, and procrastination form broadly aligned patterns or more differentiated combinations. Such information may help educators identify which aspects of adjustment warrant closer attention within a student group. Profiles remain statistical summaries whose interpretation depends on the sample, indicators, and model specification.

Person-centered research already demonstrates the relevance of studying adolescent heterogeneity (Doğrusever et al., 2026). Wilson and Matthews (2024) identified racial–mathematical identity profiles among Black American adolescents, illustrating how mathematics-related meanings combine with social identity in a particular context. Wu et al. (2024) used LPA and latent transition analysis to investigate procrastination patterns among Chinese junior high school students. These studies examine different combinations of constructs and provide methodological precedents, rather than profiles that should be reproduced in another population. Building on these strands, the present study addresses a question that these investigations do not examine: how mathematics coping capacity and identity combine with general school engagement and academic procrastination in early adolescence, and how those combinations relate to prior mathematics achievement. Its contribution lies in integrating domain-specific resources and broader academic functioning within the same analysis.

### 1.5. The Present Study

The present study examines profiles of academic resilience in mathematics, mathematics identity, school engagement, and academic procrastination among Turkish students in grades 5–8. Standardized scale total scores represent the four broad constructs. The study also examines profile differences in students’ self-reported mathematics averages from their report cards for the fall semester of the 2025–2026 academic year. These grades refer to the semester preceding the survey and serve as an external indicator of prior achievement. They are excluded from profile formation and model selection. Comparisons use the BCH approach to account for uncertainty in profile classification (Bakk & Vermunt, 2016).

Two research questions guide the analysis:

**RQ1.**  *Which profiles emerge from the joint distribution of academic resilience in mathematics, mathematics identity, school engagement, and academic procrastination?*

**RQ2.**  *To what extent do these profiles differ in students’ reported mathematics grades from the preceding semester?*

The number and composition of profiles are treated as exploratory. The design examines concurrent patterns of academic adjustment and their associations with prior achievement. It does not test the developmental ordering of the four constructs or establish causal effects on mathematics performance.

## 2. Materials and Methods

### 2.1. Research Design

This study used a cross-sectional correlational design with a person-centered analytical approach. LPA examined configurations of academic resilience in mathematics, school engagement, mathematics identity, and academic procrastination among middle school students. Academic resilience in mathematics represented students’ perceived capacity and resources for dealing with mathematics-related difficulties. School engagement and procrastination captured broader aspects of academic adjustment. Students’ previous-semester mathematics grades served as an external criterion for profile comparisons. The analyses estimated associations and did not establish temporal or causal effects.

### 2.2. Participants

Participants were recruited through convenience sampling from three public middle schools in the city center of a province in Eastern Türkiye. The analytic sample comprised 897 students, including 553 girls (61.6%) and 344 boys (38.4%). Students were 10–14 years old (M = 12.18, SD = 1.05) and attended grades 5–8. All 897 available records were retained after data screening. Table 1 presents the participant characteristics. Students’ perceived family socioeconomic status was assessed using a single self-report item asking them to classify their family’s socioeconomic status as low, medium, or high. This item reflects students’ subjective perceptions and is not an objective or comprehensive measure of family socioeconomic status.

### 2.3. Measures

One total score was calculated for each of the four scales. Totals represented broad domain composites for the profile analysis. Entering all subscales would address a more differentiated profiling question and give greater representation to constructs assessed by more subscales. The original factor structures were retained in the measurement analyses, so use of a total score did not imply that each instrument was unidimensional. The subscales informed the measurement models and supplementary analyses. Their scores were not entered alongside the corresponding totals as LPA indicators. The aim was to describe configurations of four broad observed domains. Factor-score LPA and joint factor-mixture models offer alternative representations but were not estimated in the present analysis. Therefore, the chosen specification describes observed composites without jointly modeling item-level measurement error. Current-sample CFA estimates are summarized in Table 2.

#### 2.3.1. Academic Resilience in Mathematics Scale

Academic resilience in mathematics was assessed using the Turkish form of the Academic Resilience in Mathematics Scale, developed by Ricketts et al. (2017) and adapted by Pekdemir et al. (2019). The instrument contains nine items loading on one factor. Responses range from 1 (strongly disagree) to 6 (strongly agree). Total scores range from 9 to 54, with higher scores indicating greater perceived resilience in mathematics. The Turkish adaptation reported Cronbach’s α values of 0.86 and 0.87 in its exploratory and confirmatory samples, respectively. In the present sample, α was 0.937 and ordinal ω_total_ was 0.949.

#### 2.3.2. School Engagement Scale

School engagement was measured with the School Engagement Scale developed by Fredricks et al. (2005) and adapted into Turkish by Çengel et al. (2017). The scale includes 19 items assessing behavioral engagement (5 items), emotional engagement (6 items), and cognitive engagement (8 items). Items use a five-point response format. Items 6, 11, and 18 were reverse-scored. Scores range from 19 to 95, with higher scores indicating stronger overall school engagement. The Turkish adaptation reported α = 0.89 for the full scale. In the present sample, α was 0.953 and ordinal ω_total_ was 0.972.

#### 2.3.3. Mathematics Identity Scale

Mathematics identity was assessed using the 16-item Mathematics Identity Scale developed by Satmaz and Kıncal (2023). The scale comprises interest in mathematics (7 items), recognition (6 items), and performance (3 items). The performance dimension reflects students’ perceptions of their mathematical competence and use of mathematics. Responses range from 1 (strongly disagree) to 5 (strongly agree). Total scores range from 16 to 80, with higher scores indicating a stronger mathematics identity. The development study reported α = 0.863 for the total scale. In the present sample, α was 0.948 and ordinal ω_total_ was 0.970.

#### 2.3.4. Academic Procrastination Behavior Scale

Academic procrastination was measured using the Academic Procrastination Behavior Scale for Middle School Students developed by Ocak and Karataş (2019). The scale contains 19 items across irresponsibility and preference (6 items) and environment and feelings (13 items). Items are rated on a five-point scale. Total scores range from 19 to 95, with higher scores indicating greater academic procrastination. The development study reported α = 0.946 for the full scale. In the present sample, α was 0.959 and ordinal ω_total_ was 0.976.

#### 2.3.5. Mathematics Achievement

Students reported their mathematics average from the report card issued at the end of the fall semester of the 2025–2026 academic year. Grades were recorded on a 0–100 scale and analyzed as a continuous external variable. They referred to achievement before the questionnaire assessment and were obtained through student report. The grades were not independently verified against school records. Mathematics grades were excluded from profile estimation and model selection and were subsequently compared across profiles using the BCH procedure.

### 2.4. Procedure and Ethical Considerations

Data were collected during the spring semester of the 2025–2026 academic year. The class’s designated homeroom teacher, a subject teacher assigned additional responsibility for coordinating class-related matters and parent communication, informed parents through the class social media group about the study’s purpose, voluntary participation, the scientific use of the data, and the right to withdraw at any stage. Parents communicated their permission by posting written messages in the group. Parental permissions were recorded in a list, and questionnaires were administered only to students whose parents had provided permission. Questionnaires were administered face-to-face in paper-and-pencil format during regular school hours. Students were informed about the purpose of the study, voluntary participation, confidentiality, and their right to discontinue participation. Student assent was obtained before questionnaire completion. Administration, including instructions, lasted approximately one class period. The analytic dataset did not contain personally identifying information. The study received approval from the Scientific Research Ethics Committee of Ağrı İbrahim Çeçen University on 30 April 2026 (No. 221).

### 2.5. Data Analysis

Data screening, descriptive statistics, and Pearson correlations were checked in SPSS (version 26). Initial confirmatory factor analyses and bootstrap confidence intervals for Cronbach’s α were obtained using Mplus. Item-level CFAs treated scale responses as ordered categorical variables and used WLSMV with delta parameterization. WLSMV combines diagonally weighted least-squares parameter estimation with robust standard errors and a mean- and variance-adjusted χ^2^ test (Li, 2016). Subsequent profile-model verification and secondary analyses used Mplus 8.3, as specified below. Ordinal ω and AVE were calculated from the corresponding Mplus output. SRMR was calculated from the residuals between sample-estimated and model-implied polychoric correlations, following Shi et al. (2020). The data were screened for missing values, invalid responses, identical item-response vectors, and unusual response patterns. Univariate screening used absolute z-scores greater than 3, calculated from the four scale totals using sample standard deviations. Squared Mahalanobis distances for the four totals were evaluated against a χ^2^ distribution with four degrees of freedom (*p* < 0.001). All 897 records were retained, and no imputation was required. Descriptive statistics included means, standard deviations, skewness, and kurtosis. Pearson correlations with 95% Fisher z confidence intervals summarized associations among the four scale totals and mathematics grades. Model fit was assessed using χ^2^, CFI, TLI, RMSEA with 90% confidence intervals, and polychoric SRMR. Internal consistency was evaluated using Cronbach’s α from raw item covariances and model-based ordinal ωtotal. The 95% percentile confidence intervals for α used 10,000 nonparametric bootstrap samples. Average variance extracted (AVE) was calculated for the first-order factors, and one-factor alternatives were examined for the multidimensional scales. Additional reliability and measurement results are presented in Table S2. Statistical results were checked against the corresponding software outputs; derived effect sizes and multiplicity adjustments were calculated from the saved estimates.

Initial one- through six-profile enumeration and the final five- and six-profile solutions were subsequently reproduced in Mplus 8.3. The four standardized total scores served as continuous indicators. The primary specification allowed indicator means and variances to differ across profiles and fixed within-profile indicator covariances to zero. Estimation used maximum likelihood with expectation–maximization. For the initial two- through six-profile models, 80 random starts and 80 final-stage optimizations were requested, with 20 initial-stage iterations. For the six-profile model, the search was expanded to 1000 starts and 250 final-stage optimizations, followed by 2000 starts and 500 final-stage optimizations, using 100 initial-stage iterations. Replication of the best log-likelihood was checked across starting values. BIC was the primary selection criterion, considered alongside AIC, sample-size-adjusted BIC, entropy, posterior membership probabilities, profile sizes, convergence, and interpretability (Spurk et al., 2020). This choice was informed by simulation evidence in related mixture models, in which BIC performed best among the information criteria examined and BLRT performed consistently (Nylund et al., 2007). Those findings do not guarantee the correct profile count for the present specification. Profile means and within-profile variances informed interpretation. Entropy assessed classification precision and did not determine the number of profiles alone. Parametric bootstrap likelihood ratio tests (BLRTs) comparing four versus five and five versus six profiles were run with 199 draws requested per comparison. The original-data solutions were checked against the previously replicated log-likelihoods. The reduced models used 400 initial starts and 100 final optimizations; for each generated dataset, the larger model used 2000 initial starts and 500 final optimizations (LRTSTARTS = 0 0 2000 500). Initial-stage iterations were 20 for the four-versus-five test and 100 for the five-versus-six test. Successful draws and numerical warnings were examined. Previous-semester mathematics grades were excluded from profile estimation. Their profile-specific means were compared using the BCH approach to account for classification error (Asparouhov & Muthén, 2021; Bakk & Vermunt, 2016). Manual BCH secondary models used MLR robust standard errors, with profile-specific grade variances. The secondary model included three centered grade-level indicators, with common regression slopes across profiles; adjusted means refer to the sample’s grade-level distribution. Omnibus mean equality was evaluated with a four-degree-of-freedom Wald test. Two-sided pairwise tests were evaluated at α = 0.05, applying Holm correction separately to the ten unadjusted-model contrasts and the ten grade-adjusted contrasts. Correlation *p*-values remain unadjusted. Confidence intervals are pointwise rather than simultaneous. Students were treated as independent because school and classroom identifiers were unavailable.

The omnibus effect size was a model-based eta-squared analogue, η^2^ = B/(B + W). B is the sum of the profile proportions multiplied by the squared deviations of the BCH grade means from their weighted grand mean. W is the corresponding weighted sum of the profile-specific grade variances. Estimated profile proportions, rather than modal group sizes, supplied the weights. A delta-method 95% confidence interval was derived from the robust parameter covariance matrix. Pairwise standardized differences (S) divided each BCH mean difference by the square root of the average of the two profile-specific grade variances. Their confidence intervals used the delta method in Mplus8.3. These estimates are conditional on the selected profile model and estimated BCH weights.

Sensitivity analyses compared four-, five-, and six-profile solutions under two alternative specifications: equal indicator variances across profiles with zero within-profile covariances, and profile-specific unrestricted covariance matrices. The same 897 students and four standardized indicators were used. Each model requested 2000 random starts, 500 final-stage optimizations, 100 initial-stage iterations, and up to 10,000 EM iterations, with a log-likelihood convergence criterion of 10^−7^. Fit criteria, replication of the best log-likelihood, numerical diagnostics, profile sizes, and modal membership correspondence were examined. No additional BLRT was performed for these sensitivity models.

An exploratory multinomial logistic regression used R3STEP to examine gender and perceived family socioeconomic status jointly while accounting for classification error (Asparouhov & Muthén, 2014). Girls were coded 1 and boys 0; medium and high socioeconomic status were dummy-coded against low status. Profile 5 was the reference profile. These covariates were excluded from profile formation. Odds ratios (ORs), pointwise 95% confidence intervals, and two-sided *p*-values for the logit coefficients are reported. An additional multiplicity check applied Holm correction across the 12 reported slopes (four profile contrasts × three covariates). This model did not adjust for grade level.

## 3. Results

### 3.1. Descriptive Statistics and Correlations

All 897 records were included. There were no missing values on the 63 items or mathematics grade, invalid item codes, or identical 63-item response vectors. Eleven students had an absolute standardized total score above 3 on at least one indicator. Twenty-seven students met the squared Mahalanobis-distance screening threshold for four indicators (χ^2^, df = 4, *p* < 0.001). These records were retained. The four scale totals showed limited marginal skewness (−0.186 to 0.155) and kurtosis (0.030 to 0.432). Mathematics grades ranged from 40 to 100 (M = 71.27, SD = 10.38).

Academic resilience in mathematics was positively associated with mathematics identity (r = 0.402) and school engagement (r = 0.182). Academic procrastination was negatively associated with resilience, identity, and engagement (r = −0.288, −0.219, and −0.378, respectively). Associations with mathematics grade were small, ranging from −0.066 for procrastination to 0.105 for engagement. The weakest association was between procrastination and grade (*p* = 0.048).

### 3.2. Measurement Properties

All four CFA models specifying the published factor structures converged with positive residual variances. Standardized loadings ranged from 0.780 to 0.872. CFI ranged from 0.991 to 0.997, TLI from 0.990 to 0.996, RMSEA from 0.037 to 0.047, and SRMR from 0.017 to 0.026 (Table 2). One-factor alternatives for mathematics identity, school engagement, and procrastination yielded higher RMSEA values (0.134, 0.114, and 0.133) than their original multidimensional specifications (0.037, 0.041, and 0.047). Therefore, the published dimensions were retained when evaluating the broad total-score composites. Total-score α ranged from 0.937 to 0.959, and ordinal ω_total_ ranged from 0.949 to 0.976 (Table 3). The nine first-order factors had AVE values from 0.639 to 0.727. Table S2 presents α with 95% confidence intervals, subscale reliability estimates, AVE values, and standardized loading ranges. Ordinal omega describes reliability on the underlying continuous-response metric and is distinct from reliability of the raw category sums.

### 3.3. Profile Enumeration and Classification

The five-profile model had the lowest BIC among the one- through six-profile solutions with profile-specific diagonal variances (Table 4). Compared with five profiles, six profiles reduced AIC by 14.77 and SABIC by only 0.16, but increased BIC by 28.42. The six-profile model included nine additional parameters, and BIC’s penalty for model complexity favored the more parsimonious five-profile solution. The nonsignificant five-versus-six BLRT and the substantive comparison below provided additional support for retaining five profiles within this model family. Sensitivity to alternative variance–covariance specifications is reported below and in Table S7.

**Figure 1:**
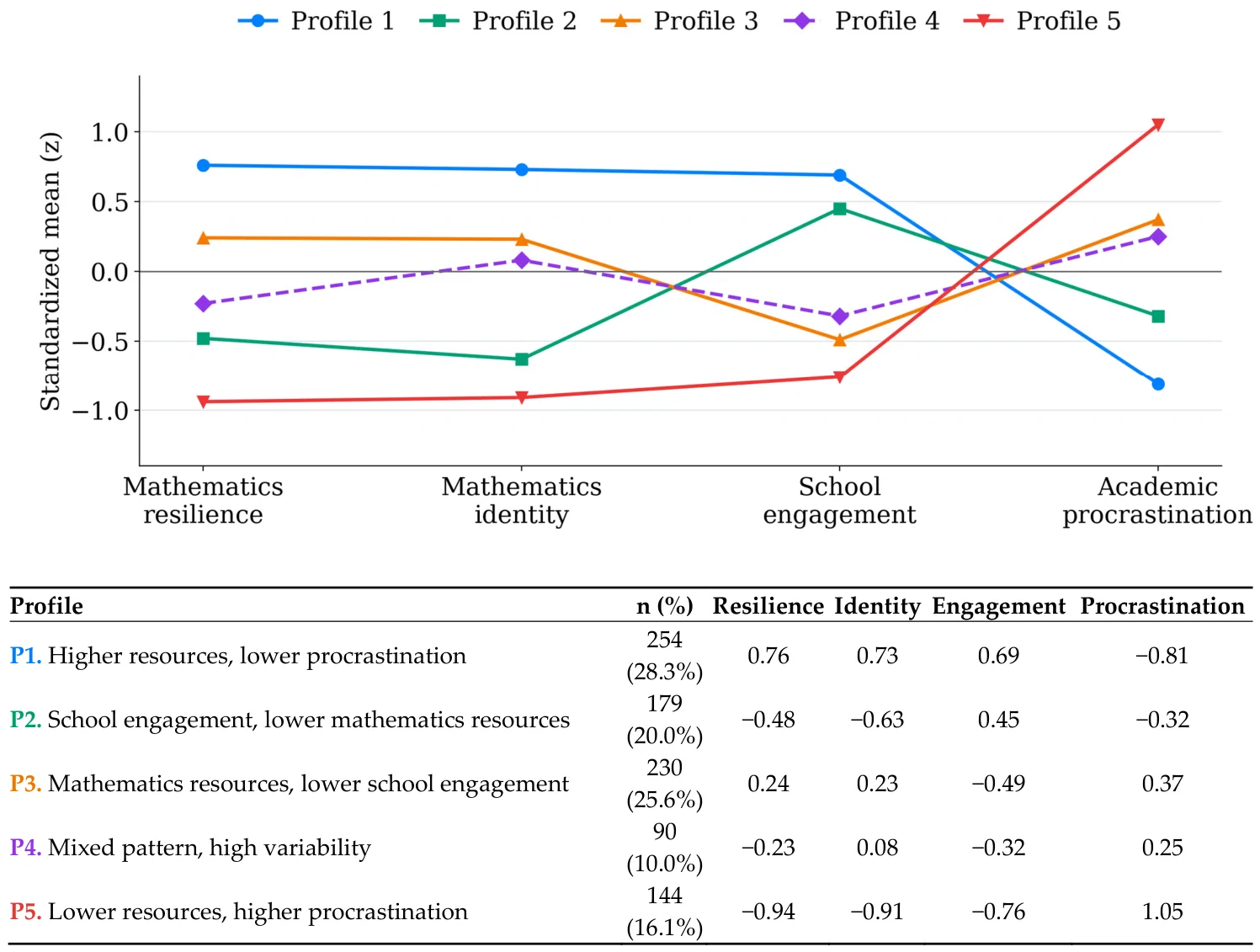
Standardized indicator means, profile labels, and modal group sizes. Note. Values are model-implied mean z-scores; counts and percentages use modal assignment. The lines connect indicators and do not represent change over time. Profile 4 has substantially greater within-profile variability (Table S3). Profile labels are descriptive and sample-relative. The figure label “mathematics resilience” denotes academic resilience in mathematics.

For the five-profile solution, the best log-likelihood (−4614.38) was obtained in 10 random-start runs and in the unperturbed run. For the six-profile solution, the best log-likelihood (−4597.99) was obtained in five random-start runs and in the unperturbed run using 2000 initial starts and 500 final-stage optimizations. The four-versus-five BLRT statistic was 153.585; approximate *p*-value of 0.0000 from 197 successful draws, with two of the 199 requested draws failing to converge. This printed value is a finite-bootstrap estimate, not a claim that the true *p*-value is zero. No best-log-likelihood replication warning was reported for these successful draws. The five-versus-six BLRT statistic was 32.770 (approximate *p* = 0.724), with all 199 draws successful and no convergence or replication warnings. Thus, this test did not support adding a sixth profile. Entropy was 0.723, and average posterior probabilities within modal groups ranged from 0.781 to 0.891. Profile 3 had the lowest average posterior probability (0.781), indicating greater classification uncertainty for this group. Modal profile sizes ranged from 90 to 254 students. Modal counts and posterior expected proportions differed, particularly for Profile 4 (10.0% versus 13.1%). Figure 1 reports modal counts and percentages. Table S3 gives the expected proportions, classification probabilities, and within-profile standard deviations. The BCH comparisons accounted for remaining classification uncertainty.

Tables S5 and S6 compare the six-profile solution with the retained five-profile solution. Six-profile classes Q5, Q3, and Q4 largely corresponded to P1, P5, and P4, respectively. Class Q1 had indicator means near the sample average (−0.24 to 0.08) and contained 181 students under modal assignment, including 91 from P2 and 71 from P3. All 85 students assigned to Q6 came from P2, whereas 149 of the 150 students assigned to Q2 came from P3. The six-profile solution therefore involved a reorganization of P2 and P3 rather than simply dividing one profile. Classification was less precise overall (entropy = 0.711 versus 0.723), and Q1 had the lowest average posterior membership probability (0.647). These descriptive comparisons use modal assignments and do not imply error-free membership.

Among the seven models summarized in Table S7, the retained five-profile diagonal model had the lowest BIC (9527.91) and SABIC (9388.18; Table S7). Within the equal-variance family, all three information criteria favored six over four or five profiles, but the six-profile solution included modal groups of 7 and 11 students, all drawn from P4. Under unrestricted covariances, BIC and SABIC favored four profiles among the obtained four- to six-profile solutions. AIC favored six, but its best log-likelihood was not replicated. The five-profile unrestricted solution was replicated only twice and also showed numerical sensitivity. These estimates therefore do not establish the best attainable fit for the more complex unrestricted models.

Profile composition also depended on the covariance assumptions. In the four-profile unrestricted model, 177 of the 179 students originally assigned to P2 and 224 of the 230 assigned to P3 were grouped together. The five-profile unrestricted model showed a similar combination and a 16-student component drawn entirely from P4. These modal comparisons are descriptive and do not account for classification error. The diagonal five-profile solution was retained as the primary representation on the basis of BIC, the BLRT within that model family, and its replicated solution; the sensitivity results do not support an invariant five-profile structure across specifications.

### 3.4. Profile Characteristics

Figure 1 presents the standardized indicator means and descriptive profile labels. Higher and lower values refer to the study sample. Profile 1 combined higher resilience, identity, and engagement with lower procrastination. Profile 2 combined stronger school engagement with lower mathematics resilience and identity. Its procrastination mean was also below the sample average. Profile 3 showed modestly higher mathematics resilience and identity alongside lower engagement and higher procrastination.

Profile 4 showed a mixed pattern with means closer to the sample average and much larger within-profile variances (2.40–2.71). Variances in the other profiles ranged from 0.25 to 0.45. The corresponding within-profile standard deviations were 1.55–1.65 for Profile 4 and 0.50–0.67 for the other profiles (Table S3). All 27 students flagged by Mahalanobis screening were assigned to Profile 4 by the modal rule. This component captured substantial dispersion and should not be interpreted as a homogeneous middle group. Profile 5 combined lower resilience, identity, and engagement with the highest procrastination mean.

The interpretation is conditional on the retained five-profile diagonal model. Replication of the best log-likelihood across starting values does not establish stability across resampled datasets or generalizability to an independent sample.

### 3.5. Profile Differences in Mathematics Achievement

The BCH analysis indicated an overall difference in mathematics grades across the five profiles, χ^2^(4) = 24.25, *p* < 0.001. Table 5 presents profile-specific mean grades after accounting for classification error. The highest mean was in Profile 1 (73.94) and the lowest in Profile 5 (67.87). These estimates refer to the student-reported 2025–2026 fall-semester report-card average. The model-based eta-squared analogue was 0.038 (delta-method 95% CI [0.008, 0.067]); between-profile mean differences represented approximately 3.8% of the model-implied total grade variance.

After Holm correction across all ten pairwise BCH contrasts (Table 6), Profile 1 had a higher mean grade than Profile 5 (difference = 6.07 points, pointwise 95% CI [3.50, 8.65], Holm-adjusted *p* < 0.001) and Profile 4 (difference = 4.11 points, pointwise 95% CI [1.25, 6.96], Holm-adjusted *p* = 0.043). The corresponding standardized differences were S = 0.55, 95% CI [0.31, 0.79], and S = 0.39, 95% CI [0.12, 0.67]. Four additional contrasts had unadjusted *p* < 0.05 but did not survive Holm correction; they are presented as exploratory. All estimates describe associations with prior achievement.

The secondary analysis adjusting for grade level gave a similar overall result, χ^2^(4) = 24.33, *p* < 0.001. Grade-adjusted means are reported in Table S4 and contrasts in Table S8. Holm correction again retained only Profile 1 versus Profile 5 (difference = 6.09 points, adjusted *p* < 0.001; S = 0.56, 95% CI [0.31, 0.80]) and Profile 1 versus Profile 4 (difference = 4.05 points, adjusted *p* = 0.043; S = 0.39, 95% CI [0.12, 0.67]). These findings do not establish causal effects.

### 3.6. Gender and Perceived Family Socioeconomic Status

The exploratory R3STEP analysis included all 897 students (Table S9). Conditional on perceived family socioeconomic status, girls had lower relative odds than boys of membership in Profile 1 versus Profile 5 (OR = 0.526, 95% CI [0.310, 0.891], *p* = 0.017), Profile 2 versus Profile 5 (OR = 0.506, 95% CI [0.277, 0.922], *p* = 0.026), and Profile 4 versus Profile 5 (OR = 0.408, 95% CI [0.207, 0.803], *p* = 0.009). The Profile 3 versus Profile 5 gender contrast was not significant (OR = 0.620, 95% CI [0.340, 1.133], *p* = 0.120). None of the eight socioeconomic-status coefficients was significant at the nominal 0.05 level (all *p* ≥ 0.307).

None of the 12 coefficients remained significant after Holm correction. The nominal gender contrasts are therefore treated as exploratory signals. Nonsignificant individual socioeconomic-status contrasts do not demonstrate equivalence between socioeconomic groups; no omnibus test of the socioeconomic-status coefficient block was reported.

## 4. Discussion

### 4.1. Overview of Findings

This study aimed to identify profiles of academic resilience in mathematics, mathematics identity, school engagement, and academic procrastination among middle school students and to examine whether these profiles differed in previous-semester mathematics achievement. Two research questions guided the investigation. The first concerned how mathematics-related resources and broader aspects of academic adjustment combined within students. The second addressed differences in students’ self-reported mathematics grades across the resulting profiles. The study approached coping with mathematics difficulties in early adolescence through students’ combined self-perceptions, school engagement, and academic behavior.

Previous person-centered research has examined racial–mathematical identity profiles among Black American adolescents (Wilson & Matthews, 2024) and patterns of academic procrastination among Chinese junior high school students (Wu et al., 2024). These studies illustrate the value of examining differences in students’ academic experiences within specific contexts. The present study extends this work by considering perceived mathematics coping capacity and identity together with general school engagement and procrastination. Its contribution concerns how these subject-specific and general characteristics combine, and how the resulting patterns relate to prior mathematics achievement.

Regarding the first research question, the retained solution comprised five profiles. Profiles 1 and 5 showed broadly aligned patterns, with higher mathematics resources and engagement accompanying lower procrastination in Profile 1 and the reverse pattern in Profile 5. Profile 2 combined greater school engagement and lower procrastination with weaker mathematics resources. Profile 3 combined modestly higher mathematics resources with lower engagement and greater procrastination. Profile 4 had means close to the sample average but considerable internal variability. These findings indicate that students’ perceived capacity to handle mathematics difficulties and their identification with mathematics can accompany different patterns of academic participation and task delay.

The aligned patterns in Profiles 1 and 5 are consistent with the correlations among the four indicators. Students’ perceived mathematics resources were positively associated with school engagement and negatively associated with procrastination. The engagement–procrastination relationship aligns with the negative association reported among Peruvian secondary students by Cutipa-Flores et al. (2025). The profiles add to this evidence by showing how several favorable or unfavorable characteristics can occur together within groups of students. They provide a basis for examining academic adjustment across beliefs, participation, and behavior while retaining the distinct meaning of each construct.

The differences between Profiles 2 and 3 offer a further contribution. In Profile 2, stronger school engagement and lower procrastination accompanied less favorable perceptions of mathematics coping capacity and identity. Finn’s (1989) participation–identification model places engagement within students’ broader connection with schooling, while situated expectancy-value theory emphasizes beliefs and meanings associated with specific academic activities (Eccles & Wigfield, 2020). This distinction helps interpret why a student may feel engaged with school yet report weaker resources as a mathematics learner. General school engagement may therefore provide an incomplete account of students’ experiences of mathematics difficulties.

In Profile 3, relatively favorable mathematics self-perceptions accompanied weaker school engagement and greater procrastination. Temporal motivation theory offers a possible explanation by considering how expected success and task value interact with the timing of rewards and action (Steel & König, 2006). Related longitudinal research also distinguishes perceived coping capacity from coping responses. Putwain and Nicholson (2026) found that coping responses predicted later academic buoyancy among older adolescents, but buoyancy did not predict later coping responses. Their study used different measures and an older sample, yet it supports considering coping beliefs and responses separately. The present pattern similarly raises questions about when mathematics-related self-perceptions accompany timely academic action.

These contrasting profiles clarify the contribution of a person-centered approach. A single ordering from better to poorer adjustment would obscure the strengths and difficulties that distinguish Profiles 2 and 3. Schnitzler et al. (2021) identified varied combinations of participation and cognitive and emotional engagement in eighth-grade mathematics lessons. Their assessment included video-coded participation within a lesson, whereas the present study combined mathematics self-perceptions with general school engagement and procrastination. Both studies support attention to variation in how academic characteristics combine. The present findings extend this perspective to the joint consideration of mathematics resources and broader academic adjustment.

The fourth profile requires separate consideration. Its near-average means masked much greater variation in individual responses, and all cases flagged by the multivariate outlier screen were assigned to this component. Bauer and Curran (2003) showed that normal mixture models can identify additional components that reflect distributional features. Therefore, Profile 4 is best interpreted as a heterogeneous component within the retained solution. Its members may have differing academic experiences, making a shared developmental explanation or a label of average adjustment premature. The broad dispersion may reflect varied combinations of resources and difficulties, response heterogeneity, or departures from the assumed within-profile distributions. The present cross-sectional results do not distinguish these possibilities or establish a transitional developmental pattern.

### 4.2. Associations with Previous Mathematics Achievement

The second research question concerned whether the identified profiles differed in previous-semester mathematics achievement. The BCH results indicated an overall difference across the five profiles, with a model-based eta-squared analogue of 0.038. The overall association was therefore limited in magnitude. After Holm correction, Profile 1 had higher estimated grades than Profiles 4 and 5; the other eight contrasts were not significant. The largest difference, between Profiles 1 and 5, was 6.07 points on the 0–100 scale and 0.55 standard-deviation units when standardized using the average within-profile variance. This selected contrast does not imply a large overall association across all five profiles.

The direction of these findings is consistent with research linking academic engagement with achievement. Wong et al.’s (2024) meta-analysis reported an average correlation of 0.33, with variation across engagement and achievement measures. In the present study, all four indicators had small correlations with mathematics grades, including a school engagement correlation of 0.105. Differences in measurement, educational setting, and the timing of assessment may contribute to the smaller association. Zero-order correlations summarize each indicator’s separate association with grades across the sample, whereas BCH contrasts compare grade means across configurations of all four indicators. Small marginal correlations and selected profile differences therefore address different comparisons. Statistical significance does not establish a large effect or improved prediction. The profile findings provide a complementary description of students’ academic experiences, but their predictive value beyond continuous scores remains to be examined.

An informative result concerns Profiles 2 and 3, whose estimated grade means were 71.41 and 71.05, respectively. Their difference was nonsignificant despite their contrasting patterns of mathematics resources, school engagement, and procrastination. Similar prior grade records can therefore accompany different current strengths and difficulties. Profile 3 had the lowest average posterior probability (0.781), so its grade estimate and its comparison with Profile 2 should be interpreted with particular attention to classification uncertainty, even when BCH correction is used. The nonsignificant comparison does not establish equivalent achievement or demonstrate that strengths in one area compensate for difficulties in another. It nevertheless highlights the value of considering students’ self-reported academic experiences alongside their grades. The comparison involving Profile 4 also requires caution because its mean represents a particularly heterogeneous group.

The interpretation of achievement differences also depends on the timing of the grade measure. Students reported grades from the semester before the survey, so the results cannot establish that their current profiles produced those grades. Earlier performance may contribute to present self-perceptions and perceived coping capacity. Putwain and Wood (2023) found that mathematics achievement predicted subsequent gains in academic buoyancy among younger students, supporting the relevance of this possibility. Longitudinal research is needed to examine whether prior performance precedes changes in academic adjustment, whether changes in these patterns precede later achievement, or whether both processes occur.

### 4.3. Theoretical and Practical Implications

The findings have theoretical and practical implications for understanding academic adjustment in early adolescence. The joint examination of the four constructs shows how mathematics-related beliefs can accompany different patterns of school engagement and academic behavior. This is relevant during a period in which adaptive coping may decline, and maladaptive responses may increase, as documented in Skinner and Saxton’s (2019) review. The findings raise questions about when perceived mathematics resources support academic action and when other barriers may be involved. The three theoretical perspectives used here help frame these questions, while their proposed mechanisms require direct testing.

In educational practice, the profiles suggest different areas to explore with students. Profile 2 points to the importance of discussing mathematics experiences even when general school engagement appears favorable. Opportunities to experience progress, feedback that makes learning visible, recognition of mathematical contributions, and activities connected with students’ interests are possible areas for support. Miller and Wang (2019) linked classroom practices with motivational beliefs and mathematics identity among sixth-grade students. Gaspard et al. (2015) also found that classroom relevance interventions strengthened mathematics value beliefs among ninth-grade students. These studies provide a basis for evaluating similar approaches with younger students who report weaker mathematics resources.

For students resembling Profile 3, attention could focus on the organization of academic action. Teachers and counselors might explore when tasks are delayed, how students begin difficult work, and what limits their school participation. Manageable study goals, explicit starting plans, and regular review of task completion are possible supports to evaluate. Students resembling Profile 5 may benefit from assessment that considers mathematics beliefs, engagement, and study behavior together, followed by coordinated support across these areas. The effectiveness of these approaches, including whether different patterns are associated with different responses to support, remains an empirical question.

The practical value of the profiles lies in guiding assessment and research on student needs. Individual decisions should also consider classroom evidence, learning histories, and students’ own accounts. This is especially important for Profile 4 because its internal variability offers limited guidance for a common intervention. The profiles should be treated as exploratory descriptions of academic experiences. Intervention studies could examine whether support informed by these combined characteristics improves engagement, task completion, or achievement and whether it adds value beyond support based on individual scale scores.

### 4.4. Limitations and Future Research

Several limitations should be considered when interpreting these findings. First, the four indicators were assessed at a single time point, limiting conclusions about the development and persistence of the profiles. The previous-semester grade provides an earlier reference point but does not make the design longitudinal. Repeated assessment would allow researchers to examine whether the patterns remain comparable over time, how students move between them, and how these transitions relate to subsequent achievement.

Second, all indicators and mathematics grades were obtained through student report. Shared response tendencies may contribute to the associations, and report-card grades were not verified against school records. Recall inaccuracies, socially desirable reporting, and differences in students’ interpretation of report-card averages may introduce error into the grade outcome. Perceived resilience was also assessed without separate measures of stress exposure or documented adversity. Therefore, the findings describe perceived resources without establishing successful adaptation under actual challenge. Future studies should combine recorded achievement and teacher reports with assessments of the difficulties students encounter and their responses to specific tasks.

Third, participants were recruited through convenience sampling from three schools in one provincial center. The profile patterns and frequencies may differ across educational settings. Shared teaching practices, peer interactions, and school climate may contribute to similarities within schools or classrooms and to the observed profile patterns and grade associations. Grade-level adjustment does not resolve this issue, and the precision of the inferential results may be affected. Studies involving more schools and retaining school and classroom identifiers would support broader generalization and appropriate analyses of clustered data.

Fourth, total scores provided concise representations of the four constructs, but totals for multidimensional scales combined information across distinct facets. Students with similar totals may differ in specific aspects of identity, engagement, or procrastination. Observed totals also retain measurement error, which may attenuate associations and affect profile estimation. CFA fit and internal consistency do not resolve this loss of detail or establish a single general factor for every scale. Measurement invariance across grade and gender groups was also not established. Future research should assess score comparability and examine whether selected subdimensions provide further insight into academic adjustment.

Fifth, the number and interpretation of profiles depend on the indicators and model assumptions. Fit criteria did not uniformly favor five profiles, and classification remained imperfect. Profile 4’s broad dispersion deserves particular attention in replication studies. The sensitivity analyses showed that P2 and P3 largely combined when within-profile covariances were estimated, and some alternatives produced small components. Numerical instability in the more complex unrestricted models further limits comparisons. The retained profiles should therefore be interpreted as a model-dependent representation rather than invariant student categories. BCH comparisons accounted for classification error, but the primary standard errors treated the estimated classification-error matrix as fixed. The reported standard errors and effect-size intervals do not propagate uncertainty from profile enumeration or estimation of the classification-error matrix. No resampling-based profile-stability claim is made. Replication in independent samples and assessment of sensitivity to distributional assumptions are still needed to determine how consistently the patterns recur (Spurk et al., 2020).

Finally, the grade comparisons were exploratory. Holm correction addressed the ten pairwise tests within each outcome model and retained two contrasts; it does not establish replication or eliminate uncertainty from model selection. Future studies should prespecify the comparisons of greatest theoretical interest and evaluate them in independent samples.

#### Gender and Perceived Family Socioeconomic Status

Gender and perceived family socioeconomic status were examined jointly in an exploratory R3STEP model, which accounted for classification error but did not adjust for grade level. Perceived family socioeconomic status was measured using one subjective item with low, medium, and high response categories, limiting inferences about objective socioeconomic conditions. None of the 12 coefficients remained significant after Holm correction. Several estimates were imprecise, and no omnibus covariate test was reported. These findings therefore do not establish equivalent profile distributions across gender or socioeconomic groups.

## 5. Conclusions

This study identified five profiles based on academic resilience in mathematics, mathematics identity, school engagement, and academic procrastination among middle school students. The profiles included broadly aligned patterns, contrasting combinations of mathematics resources and school engagement, and a heterogeneous component whose mean pattern masks substantial dispersion. The components should not be interpreted as equally distinct substantive types. After Holm correction, the higher-resource, lower-procrastination profile had higher previous-semester mathematics grades than the heterogeneous profile and the lower-resource, higher-procrastination profile; the overall association was limited in magnitude. Profiles 2 and 3 had similar estimated grades despite different combinations of strengths and difficulties. The findings contribute to understanding coping with mathematics difficulties and academic adjustment in early adolescence by examining how these characteristics occur together.

The person-centered findings encourage attention to students’ mathematics self-perceptions, participation in school, and approach to academic tasks when identifying areas for support. They offer questions for teachers and counselors to investigate with students and a basis for evaluating support responsive to differing academic needs. Replication, longitudinal assessment, and intervention research can clarify the persistence of these patterns and their usefulness for improving students’ educational experiences.

## Figures and Tables

**Table 1:** Participant characteristics.

Characteristic	Category	n	%
Gender	Girl	553	61.6
	Boy	344	38.4
Grade	5	90	10.0
	6	245	27.3
	7	337	37.6
	8	225	25.1
Age (years)	10	48	5.4
	11	185	20.6
	12	327	36.5
	13	235	26.2
	14	102	11.4
Perceived family socioeconomic status	Low	203	22.7
	Medium	507	56.5
	High	187	20.8

Note. Perceived family socioeconomic status was reported by students using one item with low, medium, and high response categories.

**Table 2:** Current-sample confirmatory factor analyses.

Measure	χ^2^ (df)	CFI/TLI	RMSEA [90% CI]	SRMR
Academic resilience in mathematics	75.82 (27)	0.997/0.996	0.045 [0.033, 0.057]	0.017
School engagement	379.03 (149)	0.991/0.990	0.041 [0.036, 0.047]	0.026
Mathematics identity	222.99 (101)	0.995/0.994	0.037 [0.030, 0.043]	0.021
Academic procrastination	455.37 (151)	0.991/0.990	0.047 [0.042, 0.052]	0.025

Note. N = 897. CFAs used WLSMV with delta parameterization for ordered categorical items. The χ^2^ tests are mean- and variance-adjusted. SRMR is the root mean square of the residuals between observed and model-implied polychoric correlations over p(p + 1)/2 unique elements, including the diagonal (p = number of items).

**Table 3:** Descriptive statistics, correlations, and total-score reliability.

Variable	1	2	3	4	5
1. Academic resilience in mathematics	—				
2. Mathematics identity	0.402 ***	—			
3. School engagement	0.182 ***	0.154 ***	—		
4. Academic procrastination	−0.288 ***	−0.219 ***	−0.378 ***	—	
5. Mathematics grade	0.100 **	0.095 **	0.105 **	−0.066 *	—
Mean	33.30	48.92	57.30	57.24	71.27
Standard deviation	8.17	10.76	12.32	12.86	10.38
Skewness	−0.16	−0.01	−0.19	0.16	0.02
Kurtosis	0.03	0.22	0.43	0.11	−0.03
Cronbach’s α	0.937	0.948	0.953	0.959	—
Ordinal ω_total_	0.949	0.970	0.972	0.976	—

Note. Entries are zero-order Pearson correlations among the four scale totals and mathematics grades. These associations are distinct from the BCH comparisons of grade means across profiles. * *p* < 0.05. ** *p* < 0.01. *** *p* < 0.001.

**Table 4:** Fit indices for latent profile solutions with profile-specific diagonal variances.

Model	AIC	BIC	SABIC	Entropy	Smallest n (%)
1 profile	10,194.30	10,232.69	10,207.29	—	897 (100.0)
2 profiles	9797.55	9879.13	9825.15	0.702	270 (30.1)
3 profiles	9590.50	9715.28	9632.71	0.703	74 (8.2)
4 profiles	9452.34	9620.31	9509.15	0.710	89 (9.9)
**5 profiles**	**9316.76**	**9527.91**	**9388.18**	**0.723**	**90 (10.0)**
6 profiles	9301.99	9556.34	9388.02	0.711	85 (9.5)

Note. Bold identifies the retained solution. AIC = Akaike information criterion. BIC = Bayesian information criterion. SABIC = sample-size-adjusted BIC. Lower information criteria indicate better relative fit. n (%) refers to the smallest modal-assignment group. Entropy is not defined for one profile. BLRT results and successful draw counts are reported in the main text.

**Table 5:** BCH-estimated mean mathematics grades by profile.

Profile	BCH Mean (SE)	95% CI
Profile 1	73.94 (0.76)	[72.45, 75.44]
Profile 2	71.41 (0.95)	[69.54, 73.27]
Profile 3	71.05 (0.81)	[69.47, 72.64]
Profile 4	69.84 (1.19)	[67.50, 72.18]
Profile 5	67.87 (1.08)	[65.75, 69.99]

Note. Grades use the 0–100 metric. All means account for profile-classification error through BCH weights. SE = standard error of the estimated mean. This primary model includes no grade-level covariate. Table S4 also reports means adjusted for grade level.

**Table 6:** Pairwise BCH comparisons of mathematics achievement.

Contrast	Difference (SE)	95% CI	Raw *p*	Holm *p*	S [95% CI]
P1–P2	2.54 (1.26)	[0.07, 5.01]	0.044	0.218	0.24 [0.01, 0.48]
P1–P3	2.89 (1.16)	[0.61, 5.17]	0.013	0.103	0.29 [0.06, 0.52]
**P1–P4**	**4.11 (1.46)**	**[1.25, 6.96]**	**0.005**	**0.043**	**0.39** **[0.12, 0.67]**
**P1–P5**	**6.07 (1.31)**	**[3.50, 8.65]**	**<0.001**	**<0.001**	**0.55** **[0.31, 0.79]**
P2–P3	0.35 (1.35)	[−2.29, 2.99]	0.795	0.968	0.04 [−0.24, 0.31]
P2–P4	1.57 (1.58)	[−1.54, 4.67]	0.323	0.968	0.16 [−0.15, 0.47]
P2–P5	3.53 (1.48)	[0.64, 6.42]	0.017	0.117	0.33 [0.06, 0.61]
P3–P4	1.22 (1.51)	[−1.75, 4.18]	0.421	0.968	0.13 [−0.19, 0.44]
P3–P5	3.18 (1.41)	[0.41, 5.95]	0.024	0.146	0.32 [0.04, 0.60]
P4–P5	1.96 (1.68)	[−1.32, 5.25]	0.241	0.964	0.19 [−0.13, 0.50]

Note. Differences are first profile minus second profile, on the 0–100 grade scale. Bold rows remain significant after Holm correction across ten contrasts. S = difference divided by the square root of the average of the two profile-specific grade variances. All 95% confidence intervals are pointwise; they are not adjusted for multiple comparisons. Raw *p*-values are two-sided robust Wald tests. Rounding follows the saved parameter estimates and standard errors.

## Data Availability

The datasets generated during and/or analysed during the current study are available from the corresponding author upon reasonable request.

## Supplementary Materials

The following supporting information can be downloaded at: https://www.mdpi.com/article/10.3390/bs16101912/s1, Table S1: Correlation results. Table S2: Reliability, average variance extracted, and standardized loading ranges. Table S3: Classification probabilities and within-profile indicator distributions. Table S4: BCH mean grades with and without grade-level adjustment. Table S5: Six-profile indicator distributions and classification. Table S6: Membership correspondence between five and six profiles. Table S7: Sensitivity to variance–covariance specification. Table S8: Grade-level-adjusted pairwise BCH comparisons. Table S9: R3STEP associations of gender and perceived family socioeconomic status with profile membership.
