# Supplementary material for: Academic Resilience in Mathematics, Mathematics Identity, School Engagement and Academic Procrastination in Early Adolescence: A Latent Profile Analysis
Source: Behav Sci (Basel). 2026 Oct 15;16(10):1912. doi: 10.3390/bs16101912 (PMC13701313; doi:10.3390/bs16101912)
Supplement: Supplementary file 1 [file behavsci-16-01912-s001.zip › behavsci-4583530-supplementary.pdf]

Supplementary Materials

These tables provide the detailed statistics supporting the main manuscript. They are included here for review and can accompany the manuscript as supplementary material.

Table S1. Correlation results.

Pair	Pearson r	95% CI for r
MR – MI	.402	[.346, .456]
MR – SE	.182	[.118, .245]
MR – AP	−.288	[−.347, −.226]
MR – Grade	.100	[.035, .165]
MI – SE	.154	[.089, .217]
MI – AP	−.219	[−.280, −.155]
MI – Grade	.095	[.030, .159]
SE – AP	−.378	[−.432, −.320]
SE – Grade	.105	[.040, .170]
AP – Grade	−.066	[−.131, −.001]

Note. N = 897. The 95% confidence intervals for Pearson r were calculated using Fisher's z transformation. MR = academic resilience in mathematics. MI = mathematics identity. SE = school engagement. AP = academic procrastination.

Table S2. Reliability, average variance extracted, and standardized loading ranges.

Scale/dimension	α [95% CI]	Ordinal ω	AVE	Loading range
**Academic resilience in mathematics**	**.937 [.930, .944]**	**.949**	**.674**	**.782–.841**
**Mathematics identity: total**	**.948 [.942, .953]**	**.970**	**—**	**.808–.865**
Interest	.921 [.912, .929]	.943	.702	.808–.865
Recognition	.912 [.902, .921]	.937	.712	.821–.860
Perceived performance	.829 [.806, .848]	.873	.696	.817–.851
**School engagement: total**	**.953 [.948, .958]**	**.972**	**—**	**.780–.858**
Behavioral	.869 [.853, .883]	.898	.639	.784–.815
Emotional	.912 [.902, .921]	.937	.712	.817–.858
Cognitive	.921 [.912, .929]	.941	.668	.780–.851
**Academic procrastination: total**	**.959 [.955, .963]**	**.976**	**—**	**.802–.872**
Irresponsibility/preference	.916 [.906, .924]	.941	.727	.834–.872
Environment/feelings	.954 [.949, .958]	.967	.692	.802–.861

Note. N = 897. Cronbach's α was calculated from raw item covariances, with 95% percentile confidence intervals based on 10,000 nonparametric bootstrap samples in the initial Mplus verification runs. For total scores, ω is ordinal ωtotal. For a first-order factor, the reported composite-reliability coefficient uses the same formula as ordinal omega. AVE was calculated as the mean squared standardized loading for each first-order factor. A single AVE is not reported for a multidimensional total. Loadings, ordinal ω, and AVE were derived from the standardized WLSMV CFA solutions. Ordinal ω for multidimensional totals incorporates correlations among first-order factors and refers to the underlying continuous responses rather than raw category sums.

Table S3. Classification probabilities and within-profile indicator distributions.

Profile	Modal n (%)	Expected %	APP	MR	MI	SE	AP
P1	254 (28.3)	27.6	.891	0.76 (0.65)	0.73 (0.55)	0.69 (0.55)	−0.81 (0.57)
P2	179 (20.0)	19.6	.806	−0.48 (0.57)	−0.63 (0.58)	0.45 (0.63)	−0.32 (0.56)
P3	230 (25.6)	24.2	.781	0.24 (0.60)	0.23 (0.56)	−0.49 (0.67)	0.37 (0.57)
P4	90 (10.0)	13.1	.891	−0.23 (1.57)	0.08 (1.65)	−0.32 (1.64)	0.25 (1.55)
P5	144 (16.1)	15.5	.854	−0.94 (0.55)	−0.91 (0.56)	−0.76 (0.52)	1.05 (0.50)

Note. Indicator cells show the model-implied mean z-score and within-profile SD in parentheses. APP = average posterior probability for the assigned profile. Expected % is calculated from summed posterior probabilities. MR = academic resilience in mathematics. MI = mathematics identity. SE = school engagement. AP = academic procrastination. The larger SDs of Profile 4 indicate greater heterogeneity around its mean pattern.

Table S4. BCH mean grades with and without grade-level adjustment.

Profile	Mean (SE)	95% CI	Adjusted mean (SE)	Adjusted 95% CI
P1	73.94 (0.76)	[72.45, 75.44]	73.94 (0.76)	[72.45, 75.43]
P2	71.41 (0.95)	[69.54, 73.27]	71.43 (0.95)	[69.56, 73.30]
P3	71.05 (0.81)	[69.47, 72.64]	71.03 (0.81)	[69.45, 72.61]
P4	69.84 (1.19)	[67.50, 72.18]	69.89 (1.18)	[67.59, 72.19]
P5	67.87 (1.08)	[65.75, 69.99]	67.85 (1.09)	[65.72, 69.98]

Table S5. Six-profile indicator distributions and classification.

Class	Modal n (%)	Expected %	APP	MR	MI	SE	AP
Q1	181 (20.2)	18.1	0.647	-0.05 (0.68)	-0.24 (0.44)	0.08 (0.46)	-0.20 (0.71)
Q2	150 (16.7)	16.9	0.775	0.27 (0.62)	0.38 (0.52)	-0.69 (0.64)	0.44 (0.56)
Q3	151 (16.8)	16.3	0.863	-0.91 (0.56)	-0.92 (0.56)	-0.76 (0.54)	1.02 (0.53)
Q4	85 (9.5)	12.8	0.909	-0.24 (1.58)	0.10 (1.66)	-0.30 (1.65)	0.26 (1.55)
Q5	245 (27.3)	26.4	0.888	0.75 (0.67)	0.78 (0.51)	0.72 (0.55)	-0.79 (0.58)
Q6	85 (9.5)	9.4	0.783	-0.58 (0.53)	-0.95 (0.49)	0.77 (0.58)	-0.32 (0.46)

Note. N = 897. Q1–Q6 follow the six-class Mplus output order and are not the P1–P5 labels used for the retained solution. Indicator cells show model-implied mean z scores and within-class SDs in parentheses. APP = average posterior probability within the modal group. Expected percentages use summed posterior probabilities. MR = academic resilience in mathematics; MI = mathematics identity; SE = school engagement; AP = academic procrastination.

Table S6. Modal membership correspondence between five and six profiles.

5-profile	Q1	Q2	Q3	Q4	Q5	Q6	Total
P1	14	0	0	1	239	0	254
P2	91	0	2	0	1	85	179
P3	71	149	5	1	4	0	230
P4	3	1	2	83	1	0	90
P5	2	0	142	0	0	0	144
Total	181	150	151	85	245	85	897

Note. Counts are based on matching all 897 records by ROWID. Rows follow the manuscript labels P1–P5; columns follow the six-profile Mplus labels Q1–Q6 in Table S5. These are modal assignments and do not account for classification uncertainty. The five-profile Mplus classes 2, 3, 4, 1, and 5 correspond to P1, P2, P3, P4, and P5, respectively.

Table S7. Sensitivity to variance–covariance specification.

Model[1]	K	Free parameters	Log likelihood	AIC	BIC	SABIC	Entropy
D	5	44	−4614.378	9316.755	9527.914	9388.177	.723
E	4	23	−4858.501	9763.002	9873.380	9800.336	.589
E	5	28	−4820.067	9696.135	9830.508	9741.585	.671
E	6	33	−4789.049	9644.098	9802.466	9697.664	.719
F	4	59	−4600.400	9318.800	9601.945	9414.571	.769
F	5	74	−4576.089	9300.177	9655.307	9420.296	.815
F	6	89	−4553.934	9285.867	9712.983	9430.335	.841

Note. N = 897. [1] D = profile-specific diagonal variances (primary model); E = equal variances across profiles, with zero covariances; F = profile-specific unrestricted covariance matrices. K = number of profiles. Lower AIC, BIC, and SABIC indicate better relative fit. The original diagonal-model enumeration is reported in Table 4. Sensitivity analyses covered K = 4–6 and do not establish optimal profile counts outside this range.

The best log-likelihood was replicated in 306, 273, and 204 final-stage runs for E4, E5, and E6, respectively, and in 189 and 2 runs for F4 and F5. It was not replicated for F6; its fit statistics are shown to document the attempted analysis, not as a verified optimum. F4, F5, and F6 had 191, 625, and 780 initial-stage runs that did not converge, and 15, 121, and 218 nonconverged final-stage runs, respectively. Information-matrix eigenvalue ratios were 4.90×10^{-4}, 2.51×10^{-7}, and 1.55×10^{-8}. All three runs reported normal termination, which did not remove the F6 replication warning.

Modal class counts were E4: 183/301/248/165; E5: 178/233/294/7/185; E6: 11/178/280/179/242/7; F4: 421/82/142/252; F5: 137/255/424/65/16; F6: 12/55/428/134/249/19 (unverified solution). Counts follow the native class order within each model and do not imply matching labels across models.

Table S8. Grade-level-adjusted pairwise BCH comparisons.

Contrast	Difference (SE)	95% CI	Raw p	Holm p	S [95% CI]
P1 – P2	2.51 (1.26)	[0.04, 4.97]	.046	.230	0.24 [0.00, 0.48]
P1 – P3	2.91 (1.16)	[0.65, 5.18]	.012	.094	0.30 [0.07, 0.52]
**P1 – P4**	**4.05 (1.44)**	**[1.24, 6.87]**	**.005**	**.043**	**0.39 [0.12, 0.67]**
**P1 – P5**	**6.09 (1.32)**	**[3.51, 8.67]**	**< .001**	**< .001**	**0.56 [0.31, 0.80]**
P2 – P3	0.41 (1.35)	[−2.24, 3.05]	.763	.977	0.04 [−0.24, 0.32]
P2 – P4	1.54 (1.57)	[−1.53, 4.62]	.326	.977	0.16 [−0.15, 0.46]
P2 – P5	3.58 (1.48)	[0.68, 6.48]	.015	.108	0.34 [0.06, 0.61]
P3 – P4	1.14 (1.50)	[−1.80, 4.07]	.447	.977	0.12 [−0.19, 0.44]
P3 – P5	3.18 (1.42)	[0.40, 5.96]	.025	.151	0.32 [0.03, 0.60]
P4 – P5	2.04 (1.67)	[−1.24, 5.31]	.222	.889	0.19 [−0.12, 0.51]

Note. N = 897. Common slopes were estimated for indicators of grades 6, 7, and 8, centered at their sample proportions; grade 5 was the reference category. Adjusted means refer to the sample's grade-level distribution. Residual grade variances were estimated separately by profile. Standardization uses the average of the two residual variances. Bold rows remain significant after Holm correction within this set of ten comparisons. Confidence intervals are pointwise. Adjusting for grade level does not account for school or classroom clustering.

Table S9. R3STEP associations of gender and perceived family socioeconomic status with profile membership.

Contrast	Predictor	β	SE	OR [95% CI]	Raw p
P1 vs P5	Girls vs boys	−0.643	0.270	0.526 [0.310, 0.891]	.017
P1 vs P5	Medium vs low SES	0.262	0.305	1.300 [0.715, 2.365]	.390
P1 vs P5	High vs low SES	−0.068	0.357	0.934 [0.464, 1.880]	.849
P2 vs P5	Girls vs boys	−0.682	0.307	0.506 [0.277, 0.922]	.026
P2 vs P5	Medium vs low SES	−0.182	0.339	0.833 [0.429, 1.619]	.590
P2 vs P5	High vs low SES	−0.413	0.404	0.662 [0.300, 1.461]	.307
P3 vs P5	Girls vs boys	−0.477	0.307	0.620 [0.340, 1.133]	.120
P3 vs P5	Medium vs low SES	0.336	0.352	1.399 [0.703, 2.787]	.339
P3 vs P5	High vs low SES	−0.150	0.422	0.861 [0.377, 1.967]	.722
P4 vs P5	Girls vs boys	−0.896	0.346	0.408 [0.207, 0.803]	.009
P4 vs P5	Medium vs low SES	0.121	0.408	1.129 [0.508, 2.509]	.767
P4 vs P5	High vs low SES	−0.056	0.476	0.946 [0.372, 2.403]	.907

Note. N = 897. All predictors were entered simultaneously. Reference categories: boys, low perceived family socioeconomic status (SES), and Profile 5. β is the unstandardized logit coefficient; OR = odds ratio. Confidence intervals are pointwise 95% intervals obtained on the logit scale and exponentiated. Raw p values are two-sided tests of the logit coefficients. None of the 12 slopes remains significant after Holm correction at $\alpha = .05$. No omnibus covariate test is reported. Results are conditional on the retained five-profile model and do not adjust for grade level. Girls: n = 553; boys: n = 344; low/medium/high SES: n = 203/507/187. Profile numbers match the main text.
